# Unveiling the causes of pericardial effusion in a contemporary case series of pericardiocentesis in Latin America

**DOI:** 10.1038/s41598-022-19339-6

**Published:** 2022-09-26

**Authors:** Juan Hernando del Portillo-Navarrete, Alejandro Pizano, Jhonattan Benavides, Andres M. Palacio, Karen Moreno-Medina, Jaime Cabrales, Darío Echeverri

**Affiliations:** 1 Department of Interventional Cardiology, Fundación Cardioinfantil-Instituto de Cardiología, Calle 163A # 13B-60, 110131 Bogotá, Colombia; 2 School of Medicine, Universidad el Bosque, Bogotá, Colombia; 3 School of Medicine, Universidad del Rosario, Bogotá, Colombia; 4 Research Department, Fundación Cardioinfantil-Instituto de Cardiología, Bogotá, Colombia

**Keywords:** Interventional cardiology, Cardiovascular diseases

## Abstract

Pericardial effusions requiring pericardiocentesis have multiple causes that vary among geographical regions and health contexts. This procedure can be performed for diagnostic or therapeutic indications. The purpose of this study was to identify the principal causes of pericardial effusions and indications for pericardiocentesis, exploring differences among groups. This was a retrospective case series of patients who underwent pericardiocentesis for pericardial effusion in a single center in Latin America. Demographic, clinical, echocardiographic, and procedural variables were recorded and analyzed. The primary outcome was to determine the causes of pericardial effusions in these patients and the indication (diagnostic, therapeutic, or both). The results are presented in two groups (inflammatory and noninflammatory) according to the cause of the pericardial effusion. One hundred sixteen patients with pericardial effusion underwent pericardiocentesis. The median age was 58 years (IQR 46.2–70.7), and 50% were male. In the noninflammatory pericardial effusion group, there were 61 cases (53%), among which neoplastic pericardial effusion was the most frequent cause (n = 25, 40.9%). In the inflammatory group, there were 55 cases (47%), and the main cause was postpericardiectomy syndrome after cardiac surgery (n = 31, 56.4%). The principal indication for pericardiocentesis was therapeutic (n = 66, 56.8%). Large pericardial effusion without hemodynamic effect of cardiac tamponade was significantly more frequent in the inflammatory group (p = 0.03). In conclusion, the principal cause of pericardial effusion in patients who underwent pericardiocentesis was postpericardiectomy syndrome after cardiac surgery, followed by neoplastic pericardial effusion. Pericardiocentesis is mainly a therapeutic procedure.

## Introduction

The pericardium is a tissue that can be affected by different systemic and cardiac illnesses. There are two phenotypic responses to a variety of injuries: acute inflammation and/or fluid accumulation^1^. The pericardium contains 10–50 mL of serous fluid between the visceral and parietal pericardium with a pressure between − 5 and 5 cmH_2_O. The increase in this fluid is known as pericardial effusion, which can produce an increase in the pericardium cavity pressure, restricting ventricular filling and decreasing cardiac output, which leads to cardiac tamponade^2,3^. Pericardiocentesis has been described since the 1970s and is a diagnostic and therapeutic procedure for treating pericardial effusion based on symptoms and echocardiographic findings^4,5^. However, according to the guidelines, the indications for invasive diagnostic techniques are some of the unmet needs in pericardial disease^2^.

Furthermore, pericardial effusion etiologies in patients who have undergone pericardiocentesis are variable for geographic regions^6–8^ and have changed over the years^9^. Therefore, these case series attempted to identify the principal causes of pericardial effusion and the indications for this procedure, exploring differences in clinical variables.

## Methods

### Study design and data collection

We present a case series of patients who underwent pericardiocentesis for pericardial effusion by interventional cardiology staff in a single center in Bogotá, Colombia between 2017 and 2018. We reviewed the database of the interventional cardiology service and selected the pericardiocentesis cases of patients over 18 years old. We classified pericardial effusion into inflammatory and noninflammatory causes based on different expert reviews^10–12^. Clinical and echocardiographic characteristics were collected based on guidelines of pericardial disease^2,3^, and procedural technical aspects and outcomes were also described^1^ (Supplementary Table 1). Patients without clinical or procedural information were excluded. The Institutional Ethics Committee (IEC) of Fundación Cardioinfantil, Instituto de Cardiología (Bogotá, Colombia) approved the study. It was carried out within the ethical principles for medical research on human beings according to the Declaration of Helsinki—59th General Assembly, Seoul, Korea, October 2008. Furthermore, informed consent waived was approved by the IEC of Fundación Cardioinfantil, Instituto de Cardiología (IEC Record No. 48-2020) due to the study's retrospective nature.

### Primary and secondary outcomes

The primary outcome was the causes of pericardial effusion in patients who required pericardiocentesis and their indications (diagnostic, therapeutic, or both). The causes were classified into two groups (inflammatory and noninflammatory), which in turn were divided into specific causes. Secondary outcomes were clinical and echocardiographic characteristics. Patients who underwent a pericardial window were identified, and their causes were also established. Success rates and procedural complications were additionally evaluated.

### Statistical analysis

The results are presented in two groups according to the cause of pericardial effusion, inflammatory and noninflammatory, and in specific causes for all the pericardiocentesis procedures conducted during the observation period. Age, weight, height, and drained liquid volume are presented as the medians and interquartile ranges. Other demographic (sex), clinical (indications and results of pericardiocentesis), and echocardiographic characteristics (effusion size and localization) are presented as absolute and relative frequencies. Fisher’s test or the χ^2^ test was used to compare clinical and echocardiographic results between the groups, and a p-value < 0.05 was considered statistically significant. SPSS v22 was used to conduct all statistical analyses.

## Results

### Clinical and demographic characteristics

A total of 116 patients with pericardial effusion underwent pericardiocentesis. The median age was 58 years (IQR 46.2–70.7), and 50% were men. The most common symptom was dyspnea (n = 50, 43.1%). Echocardiographic tamponade signs without clinical manifestations were present in 59 (50.8%) cases. Regarding pericardial effusion presentation, large pericardial effusion without a hemodynamic effect was significantly more frequent in the inflammatory group (p = 0.03), whereas cardiac tamponade with clinical manifestations was more frequent in the noninflammatory group, without significant differences. Forty-two (36.2%) patients had received anticoagulants before the procedure, with warfarin being the most frequent. There were no other significant differences among the groups when comparing the baseline clinical characteristics. Other clinical characteristics are described in Table 1.

**Table 1:** Baseline clinical characteristics of the patients.

	Pericardial effusion type	
	Inflammatory (n = 55)	Non-inflammatory (n = 61)
Male sex, no. (%)	28 (50.9)	29 (49.2)
Age years, median (IQR)	55 (40–69)	61 (49–71)
Weight kg, median (IQR)	65 (56.8–73.4)	63.7 (56–73)
Height m, (IQR)	1.64 (1.55–1.70)	1.60 (1.53–1.70)
**Signs and symptoms, n (%)**		
Asymptomatic (%)	3 (5.5)	3 (4.9)
Dyspnea	23 (41.8)	27 (44.3)
Hypotension	12 (21.8)	20 (32.8)
Tachycardia	2 (3.6)	4 (6.6)
Pericardial chest pain	21 (38.2)	10 (16.4)
Cardiac arrest, shock, syncope	2 (3.6)	3 (4.9)
**NYHA functional class, n (%)**		
I	16 (29.1)	14 (23.0)
II	26 (47.3)	23 (37.7)
III	12 (21.8)	18 (29.5)
IV	1 (1.8)	6 (9.8)
**Pericardial effusion presentation, n (%)**		
Large pericardial effusion without hemodynamic effect*	15 (27.3)	7 (11.5)
Clinical manifestations cardiac tamponade with or without aborted cardiac arrest	13 (23.6)	22 (36.1)
Echocardiographic signs of cardiac tamponade without clinical manifestations	27 (49.1)	32 (52.5)
**Other clinical characteristics, n (%)**		
Anemia	37 (67.3)	38 (62.3)
Renal replacement therapy	1 (1.8)	4 (6.6)
Previous cardiac procedures	36 (65.5)	29 (47.5)
Use of anticoagulants	20 (36.4)	22 (36.1)
**Anticoagulation therapy, n (%)**		
Unfractionated heparin	1 (1.8)	9 (14.8)
Low-molecular-weight heparin	2 (3.6)	6 (9.8)
Warfarine	15 (27.3)	6 (9.8)
Rivaroxaban	2 (3.6)	1 (1.6)

*Large pericardial effusion without hemodynamic effect was significantly more frequent in the inflammatory group (p = 0.03).

### Pericardial effusion causes in patients who underwent pericardiocentesis

#### Pericardial effusion of inflammatory causes

**Figure 1:**
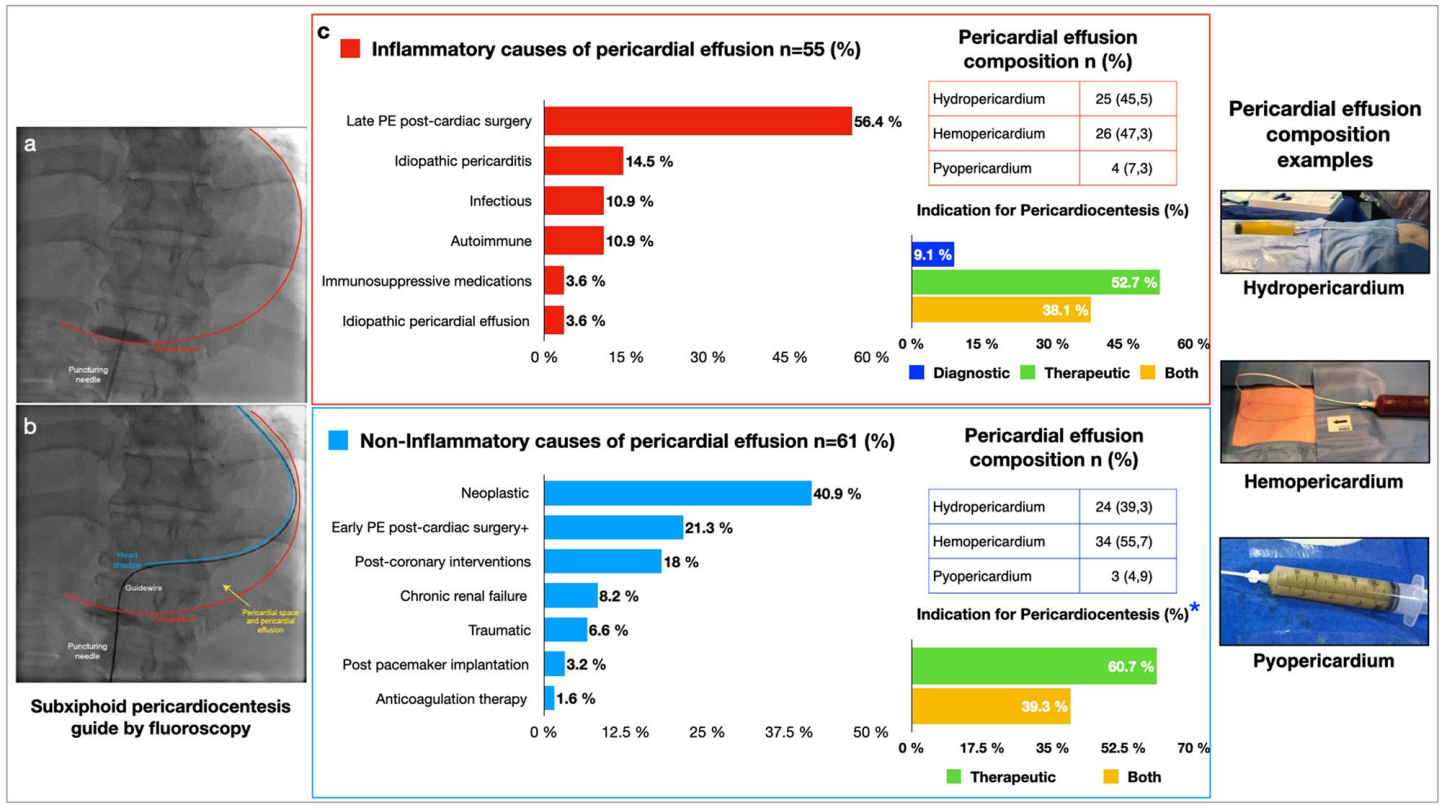
Central illustration. (**a**) Fluoroscopy guidance pericardiocentesis with the puncturing needle crossing the pericardium (red line). (**b**) Guidewire around the heart shadow (blue line) and pericardium space that contains PE between the pericardium and heart shadow (yellow arrow). (**c**) Percentages of specific causes in the inflammatory and noninflammatory groups. Percentage of diagnostic, therapeutic or both indications for pericardiocentesis in the inflammatory and noninflammatory groups. *There were no cases of only diagnostic indication for pericardiocentesis in the non-inflammatory group. Percentage of pericardial effusion composition in the groups and pericardial effusion composition examples.

Pericardial effusion of inflammatory cause was identified in 55 patients (47%). The principal etiology of inflammatory pericardial effusion was late pericardial effusion postcardiac surgery related to postpericardiectomy syndrome, which presented in 31 (56%) cases. Late pericardial effusion postcardiac surgery was defined as pericardial effusion that presented ten days after surgery, usually related to an inflammatory reaction of post pericardiectomy syndrome. The second most common etiology was idiopathic pericarditis (n = 8, 14.5%). In the infectious pericardial effusion etiologies (n = 6, 10.9%), only one case was related to *Mycobacterium tuberculosis*. Other causes are described in Table 2 and Fig. 1.

**Table 2:** Specific inflammatory and noninflammatory causes of pericardial effusion.

	n (%)
**Inflammatory causes of pericardial effusion (n = 55)**	
Late pericardial effusion postcardiac surgery*	31 (56.4)
Infectious	6 (10.9)
Autoimmune	6 (10.9)
Idiopathic pericarditis	8 (14.5)
Immunosuppressive medications	2 (3.6)
Idiopathic pericardial effusion	2 (3.6)
**Noninflammatory causes of pericardial effusion (n = 61)**	
Neoplastic	25 (40.9)
Early pericardial effusion postcardiac surgery^+^	13 (21.3)
Postcoronary interventions	11 (18.0)
Chronic renal failure	5 (8.2)
Traumatic	4 (6.6)
Post permanent or transitory pacemaker implantation	2 (3.2)
Anticoagulation therapy	1 (1.6)

*Late pericardial effusion postcardiac surgery was defined as pericardial effusion that presented ten days after surgery related to postpericardiectomy syndrome. ^+^Early pericardial effusion postcardiac surgery was defined as pericardial effusion that presented during the first ten days after surgery.

#### Pericardial effusion of noninflammatory causes

Pericardial effusion of noninflammatory cause was identified in 61 patients (53%), with neoplastic etiology being the most common cause, with 25 (40.9%) cases. Breast cancer (n = 6) followed by myelodysplastic syndromes (n = 4) were the most frequent etiologies of neoplastic pericardial effusion. Only one case was related to anticoagulation therapy. Early pericardial effusion postcardiac surgery was defined as pericardial effusion that presented during the first ten days after surgery with a possible relationship to microvascular bleeding and was classified as a noninflammatory cause (n = 13, 21.3%) (Fig. 1). The main type of cardiac surgery related to pericardial effusion was aortic valve replacement (n = 10), in which only 4 patients were classified as early pericardial effusion. Only nine patients required a pericardial window after pericardiocentesis, with persistent pericardial effusion being the principal cause.

### Echocardiographic characteristics

Most of the pericardial effusions were large (n = 70, 60.3%) and had a circumferential distribution (n = 80, 68.9%). The most frequent signs of intrapericardial pressure augmentation were diastolic collapse of the right atrium and/or right ventricle (n = 73, 62.9%) and variations in E velocities during respiration across the mitral valve and/or tricuspid valve (n = 59, 50.8%). There were no significant differences between the groups in echocardiographic findings. Additional echocardiographic findings are described in Supplementary Table 2.

### Procedural technical aspects and outcomes

The procedure was successful in 98% of the cases. There were two perforations of the cardiac chambers, one of which required emergent cardiac surgery. The most common approach was subxiphoid guided by fluoroscopy (n = 114, 98.2%). The principal indication for pericardiocentesis was therapeutic (n = 66, 56.8%) (Supplementary Table 3). There were no cases of only diagnostic indication for pericardiocentesis in the non-inflammatory group. Moreover, in the independent analyses for each indication (diagnostic, therapeutic or both), the diagnostic indication for pericardiocentesis was significantly more frequent in the inflammatory group (p = 0.04).

Pericardial effusion compositions and indications for pericardiocentesis are described in Fig. 1. There were no significant differences between the groups in pericardial effusion composition/type. Despite that, when we analyzed the composition of the pericardial effusion in each cause (Supplementary Table 4), we found hydropericardium composition was more frequent in the neoplastic cases in the non-inflammatory group. At the same time, hemopericardium was most common in post-cardiac surgery- late pericardial effusion in the inflammatory group. Intrahospital mortality was 4.3%, although none of the deaths were related to the procedure.

## Discussion

This case series identified postpericardiectomy syndrome (late pericardial effusion postcardiac surgery) as the main etiology of pericardial effusion in patients undergoing pericardiocentesis, in contrast with previous studies where the main causes reported were neoplastic, infectious, or idiopathic^7,8^. The largest registry of pericardiocentesis, published almost 20 years ago, evaluated different etiologies of pericardial effusion over the last three decades of the twentieth century in a single center. The results of that study showed a change in the causes of pericardial effusion over time, with postcardiac surgery, neoplastic, and cardiac perforations after invasive procedures being the most frequent during the nineties^9^. Our study describes similar results when evaluating specific causes, suggesting an increase in complex cardiovascular procedures.

In our study, the female sex was slightly more frequent (50.9%) compared with other series where the frequency reported of males was between 51–55%^7–9^. However, in our concept, there were no significant differences between the sex in the different case series. Furthermore, the average age between the studies is under 75 years, as in our series. Despite the causes of pericardial effusion that require pericardiocentesis varies among the regions and the period of recruitment, our case series of pericardiocentesis is comparable with others that recruited patients from other regions such as Europe, North America, and Asia^7–9,13^. Moreover, comparing the principal causes with other recent case series (last 20 years), the causes were similar (post-surgical, neoplastic, and iatrogenic)^8,13^. However, one case series from Singapore report pulmonary infection diseases as the third most common cause of the pericardial effusion^13^. While other one reported tuberculous pericarditis as their second most frequent cause of pericardial effusion^8^, this result was in China, a country with more incidence of tuberculosis^14^ than the rest of the countries with case series of pericardiocentesis in our knowledge. Additional demographic and clinical characteristics of other case series compared with ours are described in Table 3.

**Table 3:** Principal demographic and clinical characteristics of series of pericardiocentesis.

Region (*Author*, Recruitment Period)	Cases, n	Male sex, %	Age years, mean ± SD	Principal causes of pericardial effusion^ϕ^	Pericardial effusions compositions, (%)
Spain/Europe (*Sagristà-Sauleda J*, 1993–1996)^7^	126	52	56 ± 17	Idiopathic pericarditis	Not described
				Iatrogenic effusion Neoplastic	
USA/North America (*Tsang TS*, 1993–2000)*^9^	441	53	54 ± 14	Postsurgical	Hemopericardium, (51)
				Neoplastic	Hydropericardium, (44)
				Cardiac perforation from invasive procedure	Other, (5)
China/Asia (*Ma W,* 2007–2009)^8^	140	51	15–71^+^	Neoplastic Tuberculous pericarditis	Hemopericardium, (90)
				Iatrogenic	Hydropericardium, (6)
					Other, (4)
Singapore/Asia (*Cheong XP*, 2017–2019)^13^	149	54	64 ± 15	Neoplastic	Hemopericardium, (77)
				Postsurgical	Hydropericardium, (23)
				Effusion associated with pneumonia	
Colombia/South America (*Our Case series*, 2017–2018)	116	50	46–71^+^	Post cardiac surgery	Hemopericardium, (52)
				Neoplastic	Hydropericardium, (42)
				Post coronary interventions	Pyopericardium, (6,0)

*Last period of the series between 1993–2000. ^+^Age reported in interquartile range (IQR). ^ϕ^The three principal causes reported in each case series.

Our modified classification of pericardial effusion into inflammatory and noninflammatory etiologies was based on different expert reviews^10–12^; this classification allows clustering different causes that inflame, injures, or reduced lymphatic drainage of the pericardium that can result in an effusion. When comparing these two groups, we found that large pericardial effusion without hemodynamic effect was significantly more frequent in the inflammatory group, while in the noninflammatory group, cardiac tamponade with clinical manifestation was more common, but without a significant difference. This may be related to the specific etiologies in each group that could generate an acute or chronic volume overload in the pericardium cavity. This classification may help to simplify the indication and optimal timing for pericardiocentesis in patients with pericardial effusions since the hemodynamic effect was less common in the inflammatory group than in the noninflammatory group. Our results are according to a previous study where the principal causes of large pericardial effusion without tamponade were acute idiopathic pericarditis and idiopathic pericardial effusion; causes classified in our study as inflammatory causes^7^.

We also divided pericardial effusion postcardiac surgery into two types. Early pericardial effusion, which appears by the tenth postoperative day, may be related to microvascular bleeding, and late pericardial effusion is defined as pericardial effusion that presents after ten days of the procedure and is attributed to an inflammatory reaction of the pericardium^15^. This classification was based on previous studies where the maximum effusion in the postoperative state was on the tenth day after surgery^16^. In our study, the median time of pericardial effusion postcardiac surgery that required pericardiocentesis was 11.5 days (IQR = 7.0–19.7). In early and late pericardial effusion, 63% of the cases were anticoagulated, but in none of the cases was anticoagulation attributed as a cause of the effusion. However, a recent study found that postoperative anticoagulation treatment was a risk factor for subacute cardiac tamponade (defined > 48 h after cardiac surgery), a feature that could be related to these effusions^17^.

In our series, no case underwent only diagnostic pericardiocentesis in the non-inflammatory group. However, it was diagnostic and therapeutic in 39.3% of this group, compared with other studies, where the pericardiocentesis yield for diagnostic in 19%^7^.

In addition, similarly to other series^9,13^, the most frequent composition of pericardial effusion in our research was hemopericardium, followed by hydropericardium in both groups (Table 3); however, within our specific cause composition analyses, the hydropericardium was a more frequent composition in the neoplastic etiology.

The rate of complications in our study was 2%, similar to other series that are between 0.79–2%^7–9,13^, although most pericardiocentesis was guided by fluoroscopy and not by echocardiogram. The mortality was low and not related to the procedure; the pericardial effusion causes of patients who died were neoplastic (n = 4) and infectious (n = 1). In other studies, pericardial effusion caused by cancer and percutaneous cardiac interventions were related to intrahospital mortality^18,19^. The percentage of patients who needed pericardiocentesis after a percutaneous coronary intervention was 18%, and none of them had fatal outcomes during hospitalization.

The echocardiographic findings are the principal tools used to perform pericardiocentesis in patients without clinical manifestation of cardiac tamponade^11,20^, a feature that was frequent in our patients. This could be related to the specific etiologies that caused a slow accumulation of pericardial fluid, leading to cardiac tamponade only after larger volumes.

Aside from the retrospective nature of the study, a potential limitation of this data set is the limited number of patients from a single center. Moreover, we obtained the chemistry pericardial fluid analysis information in only a few cases because it is not routinely evaluated, and for that reason, it was not included. We also know that the causes of pericardial effusions in patients who underwent pericardiocentesis are variable and according to other series depend more on the geographical area. Although our institution performs a high volume of cardiovascular procedures and is located in an urban area, which could influence the results, it also admits patients from diverse regions of our country with different diseases that can compromise the pericardium.

This case series reports real data of the causes of pericardial effusion leading to pericardiocentesis in a developing country, that is comparable with developed countries. We believe that this is the largest series of pericardiocentesis published in Latin America, which changes the paradigm that has considered tuberculous pericarditis as the principal etiology of pericardial effusion in developing countries^21^, which represents only one case in our series. These findings are in accordance with the decreasing rate of tuberculosis in the world during recent decades^14^ and highlight the need for multicenter studies to elucidate the epidemiology of pericardial effusion in developing countries.

## Conclusion

Pericardiocentesis is mostly a therapeutic procedure with a low rate of complications and mortality. The pericardial effusions after cardiac surgery, related to neoplastic disease and after a percutaneous coronary intervention, were the main etiologies in patients who underwent pericardiocentesis. The inflammatory causes of pericardial effusion had milder clinical manifestations compared with noninflammatory causes.

## Supplementary Information

Supplementary Tables.
