# Publisher Correction: Unveiling the causes of pericardial effusion in a contemporary case series of pericardiocentesis in Latin America

**DOI:** 10.1038/s41598-022-23921-3

**Published:** 2022-11-14

**Authors:** Juan Hernando del Portillo-Navarrete, Alejandro Pizano, Jhonattan Benavides, Andres M. Palacio, Karen Moreno-Medina, Jaime Cabrales, Darío Echeverri

**Affiliations:** 1 Department of Interventional Cardiology, Fundación Cardioinfantil-Instituto de Cardiología, Calle 163A # 13B-60, 110131 Bogotá, Colombia; 2 School of Medicine, Universidad el Bosque, Bogotá, Colombia; 3 School of Medicine, Universidad del Rosario, Bogotá, Colombia; 4 Research Department, Fundación Cardioinfantil-Instituto de Cardiología, Bogotá, Colombia

Correction to: *Scientific Reports* https://doi.org/10.1038/s41598-022-19339-6, published online 26 September 2022

The original version of this Article contained an error in the Abstract.

“In conclusion, the principal indication for pericardiocentesis was therapeutic (n = 66, 56.8%). Large pericardial effusion without hemodynamic effect of cardiac tamponade was significantly more frequent in the inflammatory group (p = 0.03). The principal cause of pericardial effusion in patients who underwent pericardiocentesis was postpericardiectomy syndrome after cardiac surgery, followed by neoplastic pericardial effusion. Pericardiocentesis is mainly a therapeutic procedure.”

now reads:

“The principal indication for pericardiocentesis was therapeutic (n = 66, 56.8%). Large pericardial effusion without hemodynamic effect of cardiac tamponade was significantly more frequent in the inflammatory group (p = 0.03). In conclusion, the principal cause of pericardial effusion in patients who underwent pericardiocentesis was postpericardiectomy syndrome after cardiac surgery, followed by neoplastic pericardial effusion. Pericardiocentesis is mainly a therapeutic procedure.”

The original Article has been corrected.

