# Supplementary material for: Unveiling the causes of pericardial effusion in a contemporary case series of pericardiocentesis in Latin America
Source: Sci Rep. 2022 Sep 26;12:16010. doi: 10.1038/s41598-022-19339-6 (PMC9512803; doi:10.1038/s41598-022-19339-6)
Supplement: Supplementary file 1 — Supplementary Tables. [file 41598_2022_19339_MOESM1_ESM.pdf]

Supplementary Tables

Supplementary Table 1. Clinical, echocardiographic and procedural outcome definitions

Pericardial effusion presentation	
Large pericardial effusion without hemodynamic effect	Echo-free space more than 20 mm during diastole without cardiac tamponade.
Cardiac tamponade with clinical manifestations with or without aborted cardiac arrest	Cardiac tamponade: Beck's triad including hypotension (systolic blood pressure <90 mmHg), increased jugular venous pressure, and quiet heart.<br>Cardiac arrest: Unexpected circulatory arrest, occurring within 1 h of onset of acute symptoms, which is reversed by successful resuscitation maneuvers.
Echocardiographic signs of cardiac tamponade without clinical manifestations	Diastolic collapse of right chambers.<br>Variations in E velocities during respiration across the mitral valve and/or tricuspid valve, outflow that are greater than 25 and 50%.<br>Inferior vena cava (IVC) plethora (dilatation >20 mm and, 50% reduction in the diameter of IVC with respiratory phases).
**Procedural technical aspects and outcomes**	
Subxiphoid pericardiocentesis guide by fluoroscopy	The pericardium is punctured with a needle containing a contrast medium, which is slowly advanced toward the heart shadow
Pericardial effusion composition/type	Hydropericardium: Transudate; plasma ultrafiltrate

	Hemopericardium: Extension of blood into pericardial space<br>Pyopericardium: Pus (purulent fluid)

Supplementary Table 2. Echocardiographic findings

	Pericardial Effusion Type	
	**Inflammatory (n=55)**	**Non-Inflammatory (n=61)**
**Size of effusion, n (%)**		
Mild (<10 mL)	2 (3.6)	3 (4.9)
Moderate (10-20 mL)	18 (32.7)	22 (36.1)
Large (>20 mL)	35 (63.6)	35 (57.4)
**Major echocardiographic signs of cardiac tamponade, n (%)**		
Diastolic collapse of right ventricle and/or right atrium	33 (60)	40 (65.6)
Variations in E velocities during respiration across the mitral valve or tricuspid valve	24 (43.6)	35 (57.4)
Inferior vena cava (IVC) plethora*	6 (10.9)	9 (14.8)
Large pericardial effusion with swinging heart	7 (12.7)	15 (24.6)
**Localization of the pericardium, n (%)**		

Anterior	9 (16.4)	14 (23)
Posterior	6 (10.9)	2 (3.3)
Lateral	1 (1.8)	3 (4.9)
Circumferential	39 (70.9)	41 (67.2)
**Distribution, n (%)**		
Free pericadial effusion	46 (83.6)	52 (85.2)
Loculated pericadial effusion	9 (16.4)	8 (13.1)

*Dilatation >20 mm and <50% reduction in the diameter of IVC with respiratory phases

Supplementary Table 3. Pericardiocentesis procedure results

	**Pericardial Effusion Type**	
	**Inflammatory (n=55)**	**Non-Inflammatory (n=61)**
**Puncture site for pericardiocentesis, n (%)**		
Subxiphoidal	54 (98.2)	60 (98.4)
Volume drained, (SD) – mL	360 (240-465)	330 (240-430)
**Composition, n (%)**		
Hydropericardium	25 (45.5)	24 (39.3)
Hemopericardium	26 (47.3)	34 (55.7)
Pyopericardium	4 (7.3)	3 (4.9)
**Indication for pericardiocentesis, n (%)**		
Diagnostic^{+}	5 (9.1)	0
Therapeutic	29 (52.7)	37 (60.7)
Both (Diagnostic and therapeutic)	21 (38.2)	24 (39.3)

+ *Individual Fisher test analyses for the diagnostic indication for pericardiocentesis among the groups were significantly more frequent in the inflammatory group (p=0,04).*

Supplementary Table 4. Pericardial effusion composition of specific causes of pericardial effusion

	Composition, n (%)		
	**Hydropericardium**	**Hemopericardium**	**Pyopericardium**
**Specific causes of pericardial effusion**			
Infectious	4 (3,4)	1 (0,9)	2 (1,7)
Neoplastic	18 (15,5)	6 (5,2)	0
Chronic renal failure	4 (3,4)	1 (0,9)	0
Autoimmune	4 (3,4)	2 (1,7)	0
Traumatic	0	3 (2,6)	1 (0,9)
Early pericardial effusion postcardiac surgery	1 (0,9)	10 (8,6)	2 (1,7)
Late pericardial effusion postcardiac surgery	11 (9,4)	19 (16,4)	1 (0.9)
Postcoronary interventions	1 (0,9)	10 (8,6)	0
Idiopathic pericarditis	3 (2,6)	4 (3,4)	1 (0,9)

Immunosuppressive medications	0	2 (1,7)	0
Idiopathic pericardial effusion	0	2 (1,7)	0
Post permanent or transitory pacemaker implantation	0	2 (1,7)	0
Anticoagulation therapy	0	1 (0,9)	0
